# Supplementary material for: Role and mechanism of NCAPD3 in promoting malignant behaviors in gastric cancer
Source: Front Pharmacol. 2024 Apr 22;15:1341039. doi: 10.3389/fphar.2024.1341039 (PMC11070777; doi:10.3389/fphar.2024.1341039)
Supplement: Supplementary file 11 [file DataSheet2.ZIP › GSEA/immunologic/my_analysis.Gsea.1599462003817/GSE8685_IL2_STARVED_VS_IL21_ACT_IL2_STARVED_CD4_TCELL_DN.html]

Details for gene set GSE8685\_IL2\_STARVED\_VS\_IL21\_ACT\_IL2\_STARVED\_CD4\_TCELL\_DN[GSEA]

|  || Dataset | filtered\_dataset.sample\_info.cls#WT\_versus\_NCAPD3\_MUT |
| Phenotype | sample\_info.cls#WT\_versus\_NCAPD3\_MUT |
| Upregulated in class | WT |
| GeneSet | GSE8685\_IL2\_STARVED\_VS\_IL21\_ACT\_IL2\_STARVED\_CD4\_TCELL\_DN |
| Enrichment Score (ES) | 0.5228025 |
| Normalized Enrichment Score (NES) | 2.0276384 |
| Nominal p-value | 0.0052083335 |
| FDR q-value | 0.5976502 |
| FWER p-Value | 0.992 |
Table: GSEA Results Summary

  

Fig 1: Enrichment plot: GSE8685\_IL2\_STARVED\_VS\_IL21\_ACT\_IL2\_STARVED\_CD4\_TCELL\_DN      
 Profile of the Running ES Score & Positions of GeneSet Members on the Rank Ordered List

  

| SYMBOL | TITLE | RANK IN GENE LIST | RANK METRIC SCORE | RUNNING ES | CORE ENRICHMENT || 1 | 7170 | TPM3 | 24 | 1.018 | 0.0830 | Yes |
| 2 | 7571 | ZNF23 | 25 | 1.018 | 0.1831 | Yes |
| 3 | 55727 | BTBD7 | 38 | 0.954 | 0.2684 | Yes |
| 4 | 144233 | BCDIN3D | 43 | 0.942 | 0.3583 | Yes |
| 5 | 26272 | FBXO4 | 133 | 0.767 | 0.3700 | Yes |
| 6 | 29940 | DSE | 174 | 0.703 | 0.4105 | Yes |
| 7 | 54492 | NEURL1B | 177 | 0.697 | 0.4777 | Yes |
| 8 | 3304 | HSPA1B | 207 | 0.669 | 0.5228 | Yes |
| 9 | 23216 | TBC1D1 | 355 | 0.563 | 0.4728 | No |
| 10 | 54093 | SETD4 | 377 | 0.548 | 0.5117 | No |
| 11 | 9842 | PLEKHM1 | 614 | 0.416 | 0.3834 | No |
| 12 | 6301 | SARS | 856 | -0.259 | 0.2361 | No |
| 13 | 384 | ARG2 | 945 | -0.366 | 0.2091 | No |
| 14 | 3665 | IRF7 | 956 | -0.375 | 0.2388 | No |
| 15 | 222166 | MTURN | 1030 | -0.429 | 0.2287 | No |
| 16 | 91523 | PCED1B | 1041 | -0.437 | 0.2645 | No |
Table: GSEA details [plain text format]

  

Fig 2: GSE8685\_IL2\_STARVED\_VS\_IL21\_ACT\_IL2\_STARVED\_CD4\_TCELL\_DN      
 Blue-Pink O' Gram in the Space of the Analyzed GeneSet

  

Fig 3: GSE8685\_IL2\_STARVED\_VS\_IL21\_ACT\_IL2\_STARVED\_CD4\_TCELL\_DN: Random ES distribution      
 Gene set null distribution of ES for **GSE8685\_IL2\_STARVED\_VS\_IL21\_ACT\_IL2\_STARVED\_CD4\_TCELL\_DN**
